# Supplementary material for: A Highly Sensitive XNA-Based RT-qPCR Assay for the Identification of ALK, RET, and ROS1 Fusions in Lung Cancer
Source: Diagnostics (Basel). 2024 Feb 24;14(5):488. doi: 10.3390/diagnostics14050488 (PMC10930897; doi:10.3390/diagnostics14050488)
Supplement: Supplementary file 1 [file diagnostics-14-00488-s001.zip › diagnostics-2861576-supplementary.pdf]

Supplementary

A highly sensitive XNA-based RT-qPCR assay for the identification of ALK, RET, and ROS1 fusions in lung cancer.

Bongyong Lee*, Andrew Chern, Andrew Y. Fu, Aiguo Zhang and Michael Y. Sha*

DiaCarta Inc, 4385 Hopyard Rd., Suite 100, Pleasanton, CA 94588

***Corresponding author**: Michael Y. Sha: msha@diacarta.com; Bongyong Lee: blee@diacarta.com

Supplementary Table and Figure Legends

Table S1. Amsbio FFPE sample information

Table S2. Sequence information of synthetic fusion and wild type templates

Table S3. The effect of XNA on fusion detection

Figure S1. Schematic of Qfusion^{TM} ALK, RET, or ROS1 fusion detection assay targets.

Figure S2. Schematic of RT-qPCR workflow for QfusionTM ALK, RET, or ROS1 fusion detection assay.

Figure S3. XNA blocker principal and PCR cycle
XNA binds tightly to wild-type sequences with a perfect match at 70°C, effectively blocking DNA polymerase during the elongation step at 64°C. Moreover, XNA can bind to fusion sequences with imperfect matches, allowing DNA polymerase to displace XNA and amplify the target sequences.

Figure S4. Confirmation of fusion genes by Sanger sequencing.
Red arrow indicates the breaking point.

Table S1. Amsbio FFPE sample information

Sample ID	Catalog #	Patient ID	Pathology Diagnosis	Anatomical Site	Gender	Tissue Specification	Histology Grade	TNM Stage (T)	TNM Stage (N)	TNM Stage (M)	TNM Stage
190431	AMS-28011	AMS001	Adenocarcinoma	Lung	Male	Tumor	G2	T2b	Nx	Mx	IIA
190432	AMS-28011	AMS002	Large Cell Carcinoma	Lung	Male	Tumor	G2	T2b	Nx	Mx	IIA
190433	AMS-28011	AMS003	Adenocarcinoma	Lung	Female	Tumor	G2	T3	Nx	Mx	IIB
190434	AMS-28011	AMS004	Adenocarcinoma	Lung	Male	Tumor	G3	T3	Nx	Mx	IIB
190435	AMS-28011	AMS005	Adenocarcinoma	Lung	Male	Tumor	G2	T2a	Nx	Mx	IB
190436	AMS-28011	AMS006	Adenocarcinoma	Lung	Female	Tumor	G3	T2b	Nx	Mx	IIA
190437	AMS-28011	AMS007	Large Cell Carcinoma	Lung	Male	Tumor	G2	T2b	Nx	Mx	IIA
190438	AMS-28011	AMS008	Adenocarcinoma	Lung	Female	Tumor	G3	T3	Nx	Mx	IIB
190439	AMS-28011	AMS009	Adenocarcinoma	Lung	Female	Tumor	G2	T2a	Nx	Mx	IB
190222	AMS-28011	AMS010	Adenocarcinoma	Lung	Male	Tumor	G2	T2b	Nx	Mx	IIA
191573	AMS-28011	AMS011	Adenocarcinoma	Lung	Male	Tumor	G2	T2b	Nx	Mx	IIA
191574	AMS-28011	AMS012	Adenocarcinoma	Lung	Male	Tumor	G2	T2b	Nx	Mx	IIA
191575	AMS-28011	AMS013	Adenocarcinoma	Lung	Female	Tumor	G2	T2a	Nx	Mx	IB
191576	AMS-28011	AMS014	Large Cell Carcinoma	Lung	Male	Tumor	G3	T2b	Nx	Mx	IIA
191577	AMS-28011	AMS015	Large Cell Carcinoma	Lung	Male	Tumor	G3	T2b	Nx	Mx	IIA
191578	AMS-28011	AMS016	Adenocarcinoma	Lung	Male	Tumor	G2	T2a	Nx	Mx	IB
191579	AMS-28011	AMS017	Adenocarcinoma	Lung	Female	Tumor	G2	T2a	Nx	Mx	IB
191580	AMS-28011	AMS018	Large Cell Carcinoma	Lung	Male	Tumor	G3	T3	Nx	Mx	IIB
191581	AMS-28011	AMS019	Adenocarcinoma	Lung	Female	Tumor	G2	T2a	Nx	Mx	IB
191582	AMS-28011	AMS020	Adenocarcinoma	Lung	Male	Tumor	G3	T2b	Nx	Mx	IIA
191583	AMS-28011	AMS021	Adenocarcinoma	Lung	Female	Tumor	G2	T2a	Nx	Mx	IB
191584	AMS-28011	AMS022	Adenocarcinoma	Lung	Male	Tumor	G2	T1c	Nx	Mx	IA3
191585	AMS-28011	AMS023	Large Cell Carcinoma	Lung	Male	Tumor	G3	T2b	Nx	Mx	IIA
191586	AMS-28011	AMS024	Large Cell Carcinoma	Lung	Male	Tumor	G2	T2b	Nx	Mx	IIA
191587	AMS-28011	AMS025	Adenocarcinoma	Lung	Male	Tumor	G3	T3	Nx	Mx	IIB
191588	AMS-28011	AMS026	Adenocarcinoma	Lung	Female	Tumor	G2	T2b	Nx	Mx	IIA
191589	AMS-28011	AMS027	Adenocarcinoma	Lung	Male	Tumor	G2	T1c	Nx	Mx	IA3
191590	AMS-28011	AMS028	Adenocarcinoma	Lung	Male	Tumor	G2	T2a	Nx	Mx	IB
191591	AMS-28011	AMS029	Adenocarcinoma	Lung	Male	Tumor	G2	T2a	Nx	Mx	IB
191592	AMS-28011	AMS030	Adenocarcinoma	Lung	Male	Tumor	G2	T2a	Nx	Mx	IB
191593	AMS-28011	AMS031	Adenocarcinoma	Lung	Male	Tumor	G3	T2b	Nx	Mx	IIA
191594	AMS-28011	AMS032	Adenocarcinoma	Lung	Male	Tumor	G2	T1c	Nx	Mx	IA3
191595	AMS-28011	AMS033	Adenocarcinoma	Lung	Female	Tumor	G2	T2a	Nx	Mx	IB
191596	AMS-28011	AMS034	Large Cell Carcinoma	Lung	Male	Tumor	G3	T3	Nx	Mx	IIB
191597	AMS-28011	AMS035	Squamous Cell Carcinoma	Lung	Female	Tumor	G2	T2b	Nx	Mx	IIA
191598	AMS-28011	AMS036	Large Cell Carcinoma	Lung	Male	Tumor	G2	T1c	Nx	Mx	IA3
191599	AMS-28011	AMS037	Large Cell Carcinoma	Lung	Male	Tumor	G2	T2a	Nx	Mx	IB
191600	AMS-28011	AMS038	Large Cell Carcinoma	Lung	Female	Tumor	G2	T2a	Nx	Mx	IB
191601	AMS-28011	AMS039	Adenocarcinoma	Lung	Male	Tumor	G3	T2b	Nx	Mx	IIA
191602	AMS-28011	AMS040	Adenocarcinoma	Lung	Male	Tumor	G2	T2b	Nx	Mx	IIA
191603	AMS-28011	AMS041	Adenocarcinoma	Lung	Male	Tumor	G2	T2a	Nx	Mx	IB
191604	AMS-28011	AMS042	Adenocarcinoma	Lung	Male	Tumor	G2	T2b	Nx	Mx	IIA
191605	AMS-28011	AMS043	Adenocarcinoma	Lung	Male	Tumor	G3	T2a	Nx	Mx	IB
191606	AMS-28011	AMS044	Adenocarcinoma	Lung	Female	Tumor	G2	T1c	Nx	Mx	IA3
191607	AMS-28011	AMS045	Adenocarcinoma	Lung	Male	Tumor	G2	T2a	Nx	Mx	IB
191608	AMS-28011	AMS046	Adenocarcinoma	Lung	Male	Tumor	G3	T3	Nx	Mx	IIB
191609	AMS-28011	AMS047	Adenocarcinoma	Lung	Male	Tumor	G2	T2b	Nx	Mx	IIA
191610	AMS-28011	AMS048	Adenocarcinoma	Lung	Male	Tumor	G2	T2a	Nx	Mx	IB
191611	AMS-28011	AMS049	Adenocarcinoma	Lung	Male	Tumor	G3	T2b	Nx	Mx	IIA
191612	AMS-28011	AMS050	Adenocarcinoma	Lung	Male	Tumor	G2	T2a	Nx	Mx	IB
191613	AMS-28011	AMS051	Adenocarcinoma	Lung	Female	Tumor	G3	T3	Nx	Mx	IIB
191614	AMS-28011	AMS052	Adenocarcinoma	Lung	Female	Tumor	G2	T1c	Nx	Mx	IA3
191615	AMS-28011	AMS053	Adenocarcinoma	Lung	Male	Tumor	G2	T2b	Nx	Mx	IIA
191616	AMS-28011	AMS054	Adenocarcinoma	Lung	Female	Tumor	G2	T2a	Nx	Mx	IB
191617	AMS-28011	AMS055	Adenocarcinoma	Lung	Female	Tumor	G2	T2b	Nx	Mx	IIA
	T8235152	AMS056	Adenocarcinoma	Lung		Tumor					
	HP-601	AMS057		Lung		Normal					

Table S2. Sequence information of synthetic fusion and wild type templates

Name	Variants	Sequence
EML4-ALK	V1	TAATACGACTCACTATAGggCTTACTGTATGGGACTGGCAGAAGAAAGCAAAAGGAGCAGAAATAAAGACAACAAATGAAGTTGTTTTGGCTGTGGAGTTTCACCCAACAGATGCAAATACCATAATTACATGCGGTAAATCTCATATTTTCTTCTGGACCTGGAGCGGCAATTCACTAACAAGAAAACAGGGAATTTTTGGGAAATATGAAAAGCCAAAATTTGTGCAGTGTTTAGCATTCTTGGGGAATGGAGATGTTCTTACTGGAGACTCAGGTGGAGTCATGCTTATATGGAGCAAAACTACTGTAGAGCCCACACCTGGGAAAGGACCTAAAGTGTACCGCCGGAAGCACCAGGAGCTGCAAGCCATGCAGATGGAGCTGCAGAGCCCTGAGTACAAGCTGAGCAAGCTCCGCACCTCGACCATCATGACCGACTACAACCCCAACTACTGCTTTGCTGGCAAGACCTCCTCCAtatgtggatttcgagtcgtc
EML4-ALK	V2	TAATACGACTCACTATAGggTTTCATCCAAGTGGCACAGTGGTGGCCATAGGAACGCACTCAGGCAGGTGGTTTGTTCTGGATGCAGAAACCAGAGATCTAGTTTCTATCCACACAGACGGGAATGAACAGCTCTCTGTGATGCGCTACTCAATAGATGGTACCTTCCTGGCTGTAGGATCTCATGACAACTTTATTTACCTCTATGTAGTCTCTGAAAATGGAAGAAAATATAGCAGATATGGAAGGTGCACTGGACATTCCAGCTACATCACACACCTTGACTGGTCCCCAGACAACAAGTATATAATGTCTAACTCGGGAGACTATGAAATATTGTACTTGTACCGCCGGAAGCACCAGGAGCTGCAAGCCATGCAGATGGAGCTGCAGAGCCCTGAGTACAAGCTGAGCAAGCTCCGCACCTCGACCATCATGACCGACTACAACCCCAACTACTGCTTTGCTGGCAAGACCTCCTtatgtggatttcgagtcgtc
EML4-ALK	V3a	TAATACGACTCACTATAGggCAGAAAAAAAGAAAGAAAAACCACAAGGACAGAGAGAAAAAAAAGAGGAATCTCATTCTAATGATCAAAGTCCACAAATTCGAGCATCACCTTCTCCCCAGCCCTCTTCACAACCTCTCCAAATACACAGACAAACTCCAGAAAGCAAGAATGCTACTCCCACCAAAAGCATAAAACGACCATCACCAGCTGAAAAGTCACATAATTCTTGGGAAAATTCAGATGATAGCCGTAATAAATTGTCGAAAATACCTTCAACACCCAAATTAATACCAAAAGTTACCAAAACTGCAGACAAGCATAAAGATGTCATCATCAACCAAGTGTACCGCCGGAAGCACCAGGAGCTGCAAGCCATGCAGATGGAGCTGCAGAGCCCTGAGTACAAGCTGAGCAAGCTCCGCACCTCGACCATCATGACCGACTACAACCCCAACTACTGCTTTGCTGGCAAGACCTCtatgtggatttcgagtcgtc
EML4-ALK	V3b	TAATACGACTCACTATAGggGAAAGAAAAACCACAAGGACAGAGAGAAAAAAAAGAGGAATCTCATTCTAATGATCAAAGTCCACAAATTCGAGCATCACCTTCTCCCCAGCCCTCTTCACAACCTCTCCAAATACACAGACAAACTCCAGAAAGCAAGAATGCTACTCCCACCAAAAGCATAAAACGACCATCACCAGCTGAAAAGTCACATAATTCTTGGGAAAATTCAGATGATAGCCGTAATAAATTGTCGAAAATACCTTCAACACCCAAATTAATACCAAAAGTTACCAAAACTGCAGACAAGCATAAAGATGTCATCATCAACCAAGCAAAAATGTCAACTCGCGAAAAAAACAGCCAAGTGTACCGCCGGAAGCACCAGGAGCTGCAAGCCATGCAGATGGAGCTGCAGAGCCCTGAGTACAAGCTGAGCAAGCTCCGCACCTCGACCATCATGACCGACTACAACCCCAACTACTGCTTTGCTGGCAAGACCTCCTCCATCAGTGACCTGAtatgtggatttcgagtcgtc
EML4-ALK	V5a	TAATACGACTCACTATAGggCCCGGAGCCCGGCGCTTTCCCCGCAAGATGGACGGTTTCGCCGGCAGTCTCGATGATAGTATTTCTGCTGCAAGTACTTCTGATGTTCAAGATCGCCTGTCAGCTCTTGAGTCACGAGTTCAGCAACAAGAAGATGAAATCACTGTGCTAAAGGCGGCTTTGGCTGATGTTTTGAGGCGTCTTGCAATCTCTGAAGATCATGTGGCCTCAGTGAAAAAATCAGTCTCAAGTAAAGTGTACCGCCGGAAGCACCAGGAGCTGCAAGCCATGCAGATGGAGCTGCAGAGCCCTGAGTACAAGCTGAGCAAGCTCCGCACCTCGACCATCATGACCGACTACAACCCCAACTACTGCTTTGCTGGCAAGACCTCCTCCATCAGTGACCTGAAGGAGGTGCCGCGGAAAAACATCACCCTCATTCGGGGTCTGGGCCATGGAGCCTTTGGGGAGGTGTATGAAGtatgtggatttcgagtcgtc
EML4-ALK	V5b	TAATACGACTCACTATAGggGGCGCTTTCCCCGCAAGATGGACGGTTTCGCCGGCAGTCTCGATGATAGTATTTCTGCTGCAAGTACTTCTGATGTTCAAGATCGCCTGTCAGCTCTTGAGTCACGAGTTCAGCAACAAGAAGATGAAATCACTGTGCTAAAGGCGGCTTTGGCTGATGTTTTGAGGCGTCTTGCAATCTCTGAAGATCATGTGGCCTCAGTGAAAAAATCAGTCTCAAGTAAAGGTTCAGAGCTCAGGGGAGGATATGGAGATCCAGGGAGGCTTCCTGTAGGAAGTGGCCTGTGTAGTGCTTCAAGGGCCAGGCTGCCAGGCCATGTTGCAGCTGACCACCCACCTGCAGTGTACCGCCGGAAGCACCAGGAGCTGCAAGCCATGCAGATGGAGCTGCAGAGCCCTGAGTACAAGCTGAGCAAGCTCCGCACCTCGACCATCATGACCGACTACAACCCCAACTACTGCTTTGCTGGCAAGACCTCCTCCATCAGTGACCTGAAGGAGtatgtggatttcgagtcgtc
EML4-ALK	V6	TAATACGACTCACTATAGggGTGGAGTTTCACCCAACAGATGCAAATACCATAATTACATGCGGTAAATCTCATATTTTCTTCTGGACCTGGAGCGGCAATTCACTAACAAGAAAACAGGGAATTTTTGGGAAATATGAAAAGCCAAAATTTGTGCAGTGTTTAGCATTCTTGGGGAATGGAGATGTTCTTACTGGAGACTCAGGTGGAGTCATGCTTATATGGAGCAAAACTACTGTAGAGCCCACACCTGGGAAAGGACCTAAAGGAAGTGGCCTGTGTAGTGCTTCAAGGGCCAGGCTGCCAGGCCATGTTGCAGCTGACCACCCACCTGCAGTGTACCGCCGGAAGCACCAGGAGCTGCAAGCCATGCAGATGGAGCTGCAGAGCCCTGAGTACAAGCTGAGCAAGCTCCGCACCTCGACCATCATGACCGACTACAACCCCAACTACTGCTTTGCTGGCAAGACCTCCTCCATCAtatgtggatttcgagtcgtc
EML4-ALK	V7	TAATACGACTCACTATAGggGGAGCGGCAATTCACTAACAAGAAAACAGGGAATTTTTGGGAAATATGAAAAGCCAAAATTTGTGCAGTGTTTAGCATTCTTGGGGAATGGAGATGTTCTTACTGGAGACTCAGGTGGAGTCATGCTTATATGGAGCAAAACTACTGTAGAGCCCACACCTGGGAAAGGACCTAAAGGTGTATATCAAATCAGCAAACAAATCAAAGCTCATGATGGCAGTGTGTTCACACTTTGTCAGATGAGAAATGGGATGTTATTAACTGGAGGAGGGAAAGACAGAAAAATAATTCTGTGGGATCATGATCTGAATCCTGAAAGAGAAATAGAGCACCAGGAGCTGCAAGCCATGCAGATGGAGCTGCAGAGCCCTGAGTACAAGCTGAGCAAGCTCCGCACCTCGACCATCATGACCGACTACAACCCCAACTACTGCTTTGCTGGCAAGACCTCCTCCATCAGtatgtggatttcgagtcgtc
EML4-ALK	V8a	TAATACGACTCACTATAGggATCAATTTTTAGTAGGCACATCACGAAACTTTATTTTACGAGGAACATTTAATGATGGCTTCCAAATAGAAGTACAGGGTCATACAGATGAGCTTTGGGGTCTTGCCACACATCCCTTCAAAGATTTGCTCTTGACATGTGCTCAGGACAGGCAGGTGTGCCTGTGGAACTCAATGGAACACAGGCTGGAATGGACCAGGCTGGTAGATGAACCAGGACACTGTGCAGATTTTCATCCAAGTGGCACAGTGGTGGCCATAGGAACGCACTCAGGCAGGCAGGCCATGTTGCAGCTGACCACCCACCTGCAGTGTACCGCCGGAAGCACCAGGAGCTGCAAGCCATGCAGATGGAGCTGCAGAGCCCTGAGTACAAGCTGAGCAAGCTCCGCACCTCGACCATCATGACCGACTACAACCCCAACTACTGCTTTGCTGGCAAGACCTCCTCCATCAGTGACtatgtggatttcgagtcgtc
EML4-ALK	V8b	TAATACGACTCACTATAGggGGAACATTTAATGATGGCTTCCAAATAGAAGTACAGGGTCATACAGATGAGCTTTGGGGTCTTGCCACACATCCCTTCAAAGATTTGCTCTTGACATGTGCTCAGGACAGGCAGGTGTGCCTGTGGAACTCAATGGAACACAGGCTGGAATGGACCAGGCTGGTAGATGAACCAGGACACTGTGCAGATTTTCATCCAAGTGGCACAGTGGTGGCCATAGGAACGCACTCAGGCAGAGAATAATGGAAAAATAGGTCTTTAAAGATTGGCCTGTGTAGTGCTTCAAGGGCCAGGCTGCCAGGCCATGTTGCAGCTGACCACCCACCTGCAGTGTACCGCCGGAAGCACCAGGAGCTGCAAGCCATGCAGATGGAGCTGCAGAGCCCTGAGTACAAGCTGAGCAAGCTCCGCACCTCGACCATCATGACCGACTACAACCCCAACTACTGCTTTGCTGGCAAGACCTCCTCCATCAGTGACtatgtggatttcgagtcgtc
KIF5B-ALK	K15	TAATACGACTCACTATAGggTGAATCGCCTTCAAGCAGAAAATGATGCCTCTAAAGAAGAAGTGAAAGAAGTTTTACAGGCCCTAGAAGAACTTGCTGTCAATTATGATCAGAAGTCTCAGGAAGTTGAAGACAAAACTAAGGAATATGAATTGCTTAGTGATGAATTGAATCAGAAATCGGCAACTTTAGCGAGTATAGATGCTGAGCTTCAGAAACTTAAGGAAATGACCAACCACCAGAAAAAACGAGCAGCTGAGATGATGGCATCTTTACTAAAAGACCTTGCAGAAATAGGAATTGCTGTGGGAAATAATGATGTAAAGGTGTACCGCCGGAAGCACCAGGAGCTGCAAGCCATGCAGATGGAGCTGCAGAGCCCTGAGTACAAGCTGAGCAAGCTCCGCACCTCGACCATCATGACCGACTACAACCCCAACTACTGCTTTGCTGGCAAGACCTCCTCCATCAGTGACCTGAAtatgtggatttcgagtcgtc
KIF5B-ALK	K17	TAATACGACTCACTATAGggTAAAGCAGCCTGAGGGAACTGGCATGATAGATGAAGAGTTCACTGTTGCAAGACTCTACATTAGCAAAATGAAGTCAGAAGTAAAAACCATGGTGAAACGTTGCAAGCAGTTAGAAAGCACACAAACTGAGAGCAACAAAAAAATGGAAGAAAATGAAAAGGAGTTAGCAGCATGTCAGCTTCGTATCTCTCAACATGAAGCCAAAATCAAGTCATTGACTGAATACCTTCAAAATGTGGAACAAAAGAAAAGACAGTTGGAGGAATCTGTCGATGCCCTCAGTGAAGAACTAGTCCAGCTTCGAGCACAAGGTGTACCGCCGGAAGCACCAGGAGCTGCAAGCCATGCAGATGGAGCTGCAGAGCCCTGAGTACAAGCTGAGCAAGCTCCGCACCTCGACCATCATGACCGACTACAACCCCAACTACTGCTTTGCTGGCAAGACCTCCTCCATCAGTGtatgtggatttcgagtcgtc
KIF5B-ALK	K24	TAATACGACTCACTATAGggGACACCGGAGGCAGCGCTGCTCAGAAGCAAAAAATCTCCTTTCTTGAAAATAATCTTGAACAGCTCACTAAAGTGCACAAACAGTTGGTACGTGATAATGCAGATCTCCGCTGTGAACTTCCTAAGTTGGAAAAGCGACTTCGAGCTACAGCTGAGAGAGTGAAAGCTTTGGAATCAGCACTGAAAGAAGCTAAAGAAAATGCATCTCGTGATCGCAAACGCTATCAGCAAGAAGTAGATCGCATAAAGGAAGCAGTCAGGTCAAAGAATATGGCCAGAAGAGGGCATTCTGCACAGATTGTGTACCGCCGGAAGCACCAGGAGCTGCAAGCCATGCAGATGGAGCTGCAGAGCCCTGAGTACAAGCTGAGCAAGCTCCGCACCTCGACCATCATGACCGACTACAACCCCAACTACTGCTTTGCTGGCAAGACCTCCTCCATCAGTGACCTGAAGGAGGtatgtggatttcgagtcgtc
CCDC6-RET	C1;R12	TAATACGACTCACTATAGggGCATTGTCATCTCGCCGTTCCGCCTGGAGGAGCTCACCAACCGCCTGGCCTCGCTGCAGCAAGAGAACAAGGTGCTGAAGATAGAGCTGGAGACCTACAAACTGAAGTGCAAGGCACTGCAGGAGGAGAACCGCGACCTGCGCAAAGCCAGCGTGACCATCGAGGATCCAAAGTGGGAATTCCCTCGGAAGAACTTGGTTCTTGGAAAAACTCTAGGAGAAGGCGAATTTGGAAAAGTGGTCAAGGCAACGGCCTTCCATCTGAAAGGCAGAGCAGGGTACACCACGGTGGCCGTGAAGATGCTGAAAGAGAACGCCTCCCCGAGTGAGCTGCGAGACCTGCTGTCAGAGTTCAACGTCCTGAAGCAGGTCAACCACCCACATGTCATCAAtatgtggatttcgagtcgtc
CCDC6-RET	C2;R12	TAATACGACTCACTATAGggTAGAGCTGGAGACCTACAAACTGAAGTGCAAGGCACTGCAGGAGGAGAACCGCGACCTGCGCAAAGCCAGCGTGACCATCCAAGCCAGGGCTGAGCAGGAAGAAGAATTCATTAGTAACACTTTATTCAAGAAAATTCAGGCTTTGCAGAAGGAGAAAGAAACCCTTGCTGTAAATTATGAGAAAGAAGAAGAATTCCTCACTAATGAGCTCTCCAGAAAATTGATGCAGGAGGATCCAAAGTGGGAATTCCCTCGGAAGAACTTGGTTCTTGGAAAAACTCTAGGAGAAGGCGAATTTGGAAAAGTGGTCAAGGCAACGGCCTTCCATCTGAAAGGCAGAGCAGGGTACACCACGGTGGCCGTGAAGATGCTGAAAGAGAACGCCTCCCCGAGTGAGCTGCGAGACCTGCTGTCAGAGTTCAACGTCCTGAAGCAGGTCAACCACCCACATGTCATCAAtatgtggatttcgagtcgtc
KIF5B-RET	K15;R12	TAATACGACTCACTATAGggCTGTCAATTATGATCAGAAGTCTCAGGAAGTTGAAGACAAAACTAAGGAATATGAATTGCTTAGTGATGAATTGAATCAGAAATCGGCAACTTTAGCGAGTATAGATGCTGAGCTTCAGAAACTTAAGGAAATGACCAACCACCAGAAAAAACGAGCAGCTGAGATGATGGCATCTTTACTAAAAGACCTTGCAGAAATAGGAATTGCTGTGGGAAATAATGATGTAAAGGAGGATCCAAAGTGGGAATTCCCTCGGAAGAACTTGGTTCTTGGAAAAACTCTAGGAGAAGGCGAATTTGGAAAAGTGGTCAAGGCAACGGCCTTCCATCTGAAAGGCAGAGCAGGGTACACCACGGTGGCCGTGAAGATGCTGAAAGAGAACGCCTCCCCGAGTGAGCTGCGAGACCTGCTGTCAGAGTTCAACGTCCTGAAGCAGGTCAACCACCCACATGTCATCAAtatgtggatttcgagtcgtc
KIF5B-RET	K16;R12	TAATACGACTCACTATAGggTTGCAGAAATAGGAATTGCTGTGGGAAATAATGATGTAAAGCAGCCTGAGGGAACTGGCATGATAGATGAAGAGTTCACTGTTGCAAGACTCTACATTAGCAAAATGAAGTCAGAAGTAAAAACCATGGTGAAACGTTGCAAGCAGTTAGAAAGCACACAAACTGAGAGCAACAAAAAAATGGAAGAAAATGAAAAGGAGTTAGCAGCATGTCAGCTTCGTATCTCTCAAGAGGATCCAAAGTGGGAATTCCCTCGGAAGAACTTGGTTCTTGGAAAAACTCTAGGAGAAGGCGAATTTGGAAAAGTGGTCAAGGCAACGGCCTTCCATCTGAAAGGCAGAGCAGGGTACACCACGGTGGCCGTGAAGATGCTGAAAGAGAACGCCTCCCCGAGTGAGCTGCGAGACCTGCTGTCAGAGTTCAACGTCCTGAAGCAGGTCAACCACCCACATGTCATCAAtatgtggatttcgagtcgtc
KIF5B-RET	K22;R12	TAATACGACTCACTATAGggACCAGAAAATGATGTTAGAGCAGGAACGTCTAAGAGTAGAACATGAGAAGTTGAAAGCCACAGATCAGGAAAAGAGCAGAAAACTACATGAACTTACGGTTATGCAAGATAGACGAGAACAAGCAAGACAAGACTTGAAGGGTTTGGAAGAGACAGTGGCAAAAGAACTTCAGACTTTACACAACCTGCGCAAACTCTTTGTTCAGGACCTGGCTACAAGAGTTAAAAAGGAGGATCCAAAGTGGGAATTCCCTCGGAAGAACTTGGTTCTTGGAAAAACTCTAGGAGAAGGCGAATTTGGAAAAGTGGTCAAGGCAACGGCCTTCCATCTGAAAGGCAGAGCAGGGTACACCACGGTGGCCGTGAAGATGCTGAAAGAGAACGCCTCCCCGAGTGAGCTGCGAGACCTGCTGTCAGAGTTCAACGTCCTGAAGCAGGTCAACCACCCACATGTCATCAAtatgtggatttcgagtcgtc
KIF5B-RET	K23;R12	TAATACGACTCACTATAGggAAGATAGACGAGAACAAGCAAGACAAGACTTGAAGGGTTTGGAAGAGACAGTGGCAAAAGAACTTCAGACTTTACACAACCTGCGCAAACTCTTTGTTCAGGACCTGGCTACAAGAGTTAAAAAGAGTGCTGAGATTGATTCTGATGACACCGGAGGCAGCGCTGCTCAGAAGCAAAAAATCTCCTTTCTTGAAAATAATCTTGAACAGCTCACTAAAGTGCACAAACAGGAGGATCCAAAGTGGGAATTCCCTCGGAAGAACTTGGTTCTTGGAAAAACTCTAGGAGAAGGCGAATTTGGAAAAGTGGTCAAGGCAACGGCCTTCCATCTGAAAGGCAGAGCAGGGTACACCACGGTGGCCGTGAAGATGCTGAAAGAGAACGCCTCCCCGAGTGAGCTGCGAGACCTGCTGTCAGAGTTCAACGTCCTGAAGCAGGTCAACCACCCACATGTCATCAAtatgtggatttcgagtcgtc
NCOA4-RET	N8;R12	TAATACGACTCACTATAGggTTCCTGAGCACTTGATGGCTCATGCTAGTTCAGCAAATATTGGGCCCTTCCTGGAGAAGAGAGGCTGTATCTCCATGCCAGAGCAGAAGTCAGCATCCGGTATTGTAGCTGTCCCTTTCAGCGAATGGCTCCTTGGAAGCAAACCTGCCAGTGGTTATCAAGCTCCTTACATACCCAGCACCGACCCCCAGGACTGGCTTACCCAAAAGCAGACCTTGGAGAACAGTCAGGAGGATCCAAAGTGGGAATTCCCTCGGAAGAACTTGGTTCTTGGAAAAACTCTAGGAGAAGGCGAATTTGGAAAAGTGGTCAAGGCAACGGCCTTCCATCTGAAAGGCAGAGCAGGGTACACCACGGTGGCCGTGAAGATGCTGAAAGAGAACGCCTCCCCGAGTGAGCTGCGAGACCTGCTGTCAGAGTTCAACGTCCTGAAGCAGGTCAACCACCCACATGTCATCAAtatgtggatttcgagtcgtc
SDC4-ROS1	S2;R32	TAATACGACTCACTATAGggAGCCTGCGCGCCTTCTCCAGTCCGCGGTGCCATGGCCCCCGCCCGTCTGTTCGCGCTGCTGCTGTTCTTCGTAGGCGGAGTCGCCGAGTCGATCCGAGAGACTGAGGTCATCGACCCCCAGGACCTCCTAGAAGGCCGATACTTCTCCGGAGCCCTACCAGACGATGAGGATGTAGTGGGGCCCGGGCAGGAATCTGATGACTTTGAGCTGTCTGGCTCTGGAGATCTGGCTGGAGTCCCAAATAAACCAGGCATTCCCAAATTACTAGAAGGGAGTAAAAATTCAATACAGTGGGAGAAAGCTGAAGATAATGGATGTAGAATTACATACTATATCCTTGAGATAAGAAAGAGCACTTCAAATAATTTACAGAACCAGAATTTAAGGTGGAAGATGACATTTAATGGATCCTGCAGTAGTGTTTGCACATGGAAGTCCAAAAACCTGAAAGGAATATTTtatgtggatttcgagtcgtc
SLC34A2-ROS1	S4;R32	TAATACGACTCACTATAGggTCTGCCGTCCTACTCCACGGCTACACTGATAGATGAGCCCACTGAGGTGGATGACCCCTGGAACCTACCCACTCTTCAGGACTCGGGGATCAAGTGGTCAGAGAGAGACACCAAAGGGAAGATTCTCTGTTTCTTCCAAGGGATTGGGAGATTGATTTTACTTCTCGGATTTCTCTACTTTTTCGTGTGCTCCCTGGATATTCTTAGTAGCGCCTTCCAGCTGGTTGGAGCTGGAGTCCCAAATAAACCAGGCATTCCCAAATTACTAGAAGGGAGTAAAAATTCAATACAGTGGGAGAAAGCTGAAGATAATGGATGTAGAATTACATACTATATCCTTGAGATAAGAAAGAGCACTTCAAATAATTTACAGAACCAGAATTTAAGGTGGAAGATGACATTTAATGGATCCTGCAGTAGTGTTTGCACATGGAAGTCCAAAAACCTGAAAGGAATATTTtatgtggatttcgagtcgtc
SLC34A2-ROS1	S13;R32	TAATACGACTCACTATAGggAGATGCGCTGCTGCTGCTGCTGCCGCGTGTGCTGCCGCGCGTGCTGCTTGCTGTGTGGCTGCCCCAAGTGCTGCCGCTGCAGCAAGTGCTGCGAGGACTTGGAGGAGGCGCAGGAGGGGCAGGATGTCCCTGTCAAGGCTCCTGAGACCTTTGATAACATAACCATTAGCAGAGAGGCTCAGGGTGAGGTCCCTGCCTCGGACTCAAAGACCGAATGCACGGCCTTGTAGCTGGAGTCCCAAATAAACCAGGCATTCCCAAATTACTAGAAGGGAGTAAAAATTCAATACAGTGGGAGAAAGCTGAAGATAATGGATGTAGAATTACATACTATATCCTTGAGATAAGAAAGAGCACTTCAAATAATTTACAGAACCAGAATTTAAGGTGGAAGATGACATTTAATGGATCCTGCAGTAGTGTTTGCACATGGAAGTCCAAAAACCTGAAAGGAATATTTtatgtggatttcgagtcgtc
CD74-ROS1	C6;R34	TAATACGACTCACTATAGggCAAGTATGGCAACATGACAGAGGACCATGTGATGCACCTGCTCCAGAATGCTGACCCCCTGAAGGTGTACCCGCCACTGAAGGGGAGCTTCCCGGAGAACCTGAGACACCTTAAGAACACCATGGAGACCATAGACTGGAAGGTCTTTGAGAGCTGGATGCACCATTGGCTCCTGTTTGAAATGAGCAGGCACTCCTTGGAGCAAAAGCCCACTGACGCTCCACCGAAAGATGATTTTTGGATACCAGAAACAAGTTTCATACTTACTATTATAGTTGGAATATTTCTGGTTGTTACAATCCCACTGACCTTTGTCTGGCATAGAAGATTAAAGAATCAAAAAAGTGCCAAGGAAGGGGTGACAGTGCTTATAAACGAAGACAAAGAGTTGGCTGAGCTGCGAGGTCTGGCAGCCGGAGTAGGCCTGGCTAATGCCTGCTATGCAATACATACTCTTCCAtatgtggatttcgagtcgtc
EZR-ROS1	E10;R34	TAATACGACTCACTATAGggCCATGAGTTGTATATGCGCCGCAGGAAGCCTGACACCATCGAGGTGCAGCAGATGAAGGCCCAGGCCCGGGAGGAGAAGCATCAGAAGCAGCTGGAGCGGCAACAGCTGGAAACAGAGAAGAAAAGGAGAGAAACCGTGGAGAGAGAGAAAGAGCAGATGATGCGCGAGAAGGAGGAGTTGATGCTGCGGCTGCAGGACTATGAGGAGAAGACAAAGAAGGCAGAGAGAGATGATTTTTGGATACCAGAAACAAGTTTCATACTTACTATTATAGTTGGAATATTTCTGGTTGTTACAATCCCACTGACCTTTGTCTGGCATAGAAGATTAAAGAATCAAAAAAGTGCCAAGGAAGGGGTGACAGTGCTTATAAACGAAGACAAAGAGTTGGCTGAGCTGCGAGGTCTGGCAGCCGGAGTAGGCCTGGCTAATGCCTGCTATGCAATACATACTCTTCCAtatgtggatttcgagtcgtc
GOPC-ROS1	G8;R35	TAATACGACTCACTATAGggCCTAAGGGACACAAAGCATAAAGAAGCTGTAACTATTCTTTCTCAGCAGAGAGGAGAGATTGAATTTGAAGTAGTTTATGTGGCTCCTGAAGTGGATTCTGATGATGAAAACGTAGAGTATGAAGATGAGAGTGGACATCGTTACCGTTTGTACCTTGATGAGTTAGAAGGAGGTGGTAACCCTGGTGCTAGTTGCAAAGACACAAGTGGGGAAATCAAAGTATTACAAGTCTGGCATAGAAGATTAAAGAATCAAAAAAGTGCCAAGGAAGGGGTGACAGTGCTTATAAACGAAGACAAAGAGTTGGCTGAGCTGCGAGGTCTGGCAGCCGGAGTAGGCCTGGCTAATGCCTGCTATGCAATACATACTCTTCCAACCCAAGAGGAGATTGAAAATCTTCCTGCCTTCCCTCGGGAAAAACTGACTCTGCGTCTCTTGCTGGGAAGTGGAGCCTTTGGAtatgtggatttcgagtcgtc
GOPC-ROS1	G4;R36	TAATACGACTCACTATAGggGCTGACTCTGGTACCATTAAGGCAAAATTGTCTGGCCCCTCTGTGGAGGAGCTGGAAAGAGAGCTTGAGGCAAACAAAAAAGAAAAAATGAAAGAAGCACAACTTGAAGCTGAAGTGAAATTGTTGAGAAAAGAGAATGAAGCCCTTCGTAGACATATAGCTGTTCTCCAGGCTGAAGTATATGGGGCGAGACTAGCTGCCAAGTACTTGGATAAGGAACTGGCAGGAAGTACTCTTCCAACCCAAGAGGAGATTGAAAATCTTCCTGCCTTCCCTCGGGAAAAACTGACTCTGCGTCTCTTGCTGGGAAGTGGAGCCTTTGGAGAAGTGTATGAAGGAACAGCAGTGGACATCTTAGGAGTTGGAAGTGGAGAAATCAAAGTAGCAGTGAAGACTTTGAAGAAGGGTTCCACAGACCAGGAGAAGATTGAATTCCTGAAGGAGGCACATCTGATGAGCAtatgtggatttcgagtcgtc
ALK		TAATACGACTCACTATAGggATCCTGTACACCCCAGCTTTAAAAGTGATGGAAGGCCACGGGGAAGTGAATATTAAGCATTATCTAAACTGCAGTCACTGTGAGGTAGACGAATGTCACATGGACCCTGAAAGCCACAAGGTCATCTGCTTCTGTGACCACGGGACGGTGCTGGCTGAGGATGGCGTCTCCTGCATTGTGTCACCCACCCCGGAGCCACACCTGCCACTCTCGCTGATCCTCTCTGTGGTGACCTCTGCCCTCGTGGCCGCCCTGGTCCTGGCTTTCTCCGGCATCATGATTGTGTACCGCCGGAAGCACCAGGAGCTGCAAGCCATGCAGATGGAGCTGCAGAGCCCTGAGTACAAGCTGAGCAAGCTCCGCACCTCGACCATCATGACCGACTACAACCCCAACTACTGCTTTGCTGGCAAGACCTCCTCCATCAGTGACCTGAAGGAGGTGCCGCGGAAAAACATCtatgtggatttcgagtcgtc
RET		TAATACGACTCACTATAGggCGAAGACATCCAGGATCCACTGTGCGACGAGCTGTGCCGCACGGTGATCGCAGCCGCTGTCCTCTTCTCCTTCATCGTCTCGGTGCTGCTGTCTGCCTTCTGCATCCACTGCTACCACAAGTTTGCCCACAAGCCACCCATCTCCTCAGCTGAGATGACCTTCCGGAGGCCCGCCCAGGCCTTCCCGGTCAGCTACTCCTCTTCCGGTGCCCGCCGGCCCTCGCTGGACTCCATGGAGAACCAGGTCTCCGTGGATGCCTTCAAGATCCTGGAGGATCCAAAGTGGGAATTCCCTCGGAAGAACTTGGTTCTTGGAAAAACTCTAGGAGAAGGCGAATTTGGAAAAGTGGTCAAGGCAACGGCCTTCCATCTGAAAGGCAGAGCAGGGTACACCACGGTGGCCGTGAAGATGCTGAAAGAGAACGCCTCCCCGAGTGAGCTGCGAGACCTGCTGTCAGAtatgtggatttcgagtcgtc
ROS1		TAATACGACTCACTATAGggATGTTAAAACTTCATGCAGCCAAGGTCCTGCTTATGTCTGTAATATCACAAATCTACAACCTTATACTTCATATAATGTCAGAGTAGTGGTGGTTTATAAGACGGGAGAAAATAGCACCTCACTTCCAGAAAGCTTTAAGACAAAAGCTGGAGTCCCAAATAAACCAGGCATTCCCAAATTACTAGAAGGGAGTAAAAATTCAATACAGTGGGAGAAAGCTGAAGATAATGGATGTAGAATTACATACTATATCCTTGAGATAAGAAAGAGCACTTCAAATAATTTACAGAACCAGAATTTAAGGTGGAAGATGACATTTAATGGATCCTGCAGTAGTGTTTGCACATGGAAGTCCAAAAACCTGAAAGGAATATTTCAGTTCAGAGTAGTAGCTGCAAATAATCTAGGGTTTGGTGAATATAGTGGAATCAGTGAGAATATTATATTAGTTGGAGATGATTTTTGGATACCAGAAACAAGTTTCATACTTACTATTATAGTTGGAATATTTCTGGTTGTTACAATCCCACTGACCTTTGTCTGGCATAGAAGATTAAAGAATCAAAAAAGTGCCAAGGAAGGGGTGACAGTGCTTATAAACGAAGACAAAGAGTTGGCTGAGCTGCGAGGTCTGGCAGCCGGAGTAGGCCTGGCTAATGCCTGCTATGCAATACATACTCTTCCAACCCAAGAGGAGATTGAAAATCTTCCTGCCTTCCCTCGGGAAAAACTGACTCTGCGTCTCTTGCTGGGAAGTGGAGCCTTTGGAGAAGTGTATGAAGGAACAGCAGTGGACATCtatgtggatttcgagtcgtc

Table S3. The effect of XNA on fusion detection

EML4-ALK V1	50 copies	
	no XNA	XNA
**Cq Rep 1**	31.04	31.57
**Cq Rep 2**	30.69	30.84
**Cq Rep 3**	30.86	31.14
**Cq Rep 4**	31.01	30.95
**Cq Rep 5**	31.04	31.12
**Cq Rep 6**	31.10	31.15
**Cq Rep 7**	30.99	31.36
**Cq Rep 8**	30.99	31.25
**Cq Rep 9**	31.06	31.35
**Cq Rep 10**	30.84	31.15
**AVG**	30.96	31.19
**SD**	0.13	0.21
**Delta Cq**		0.22

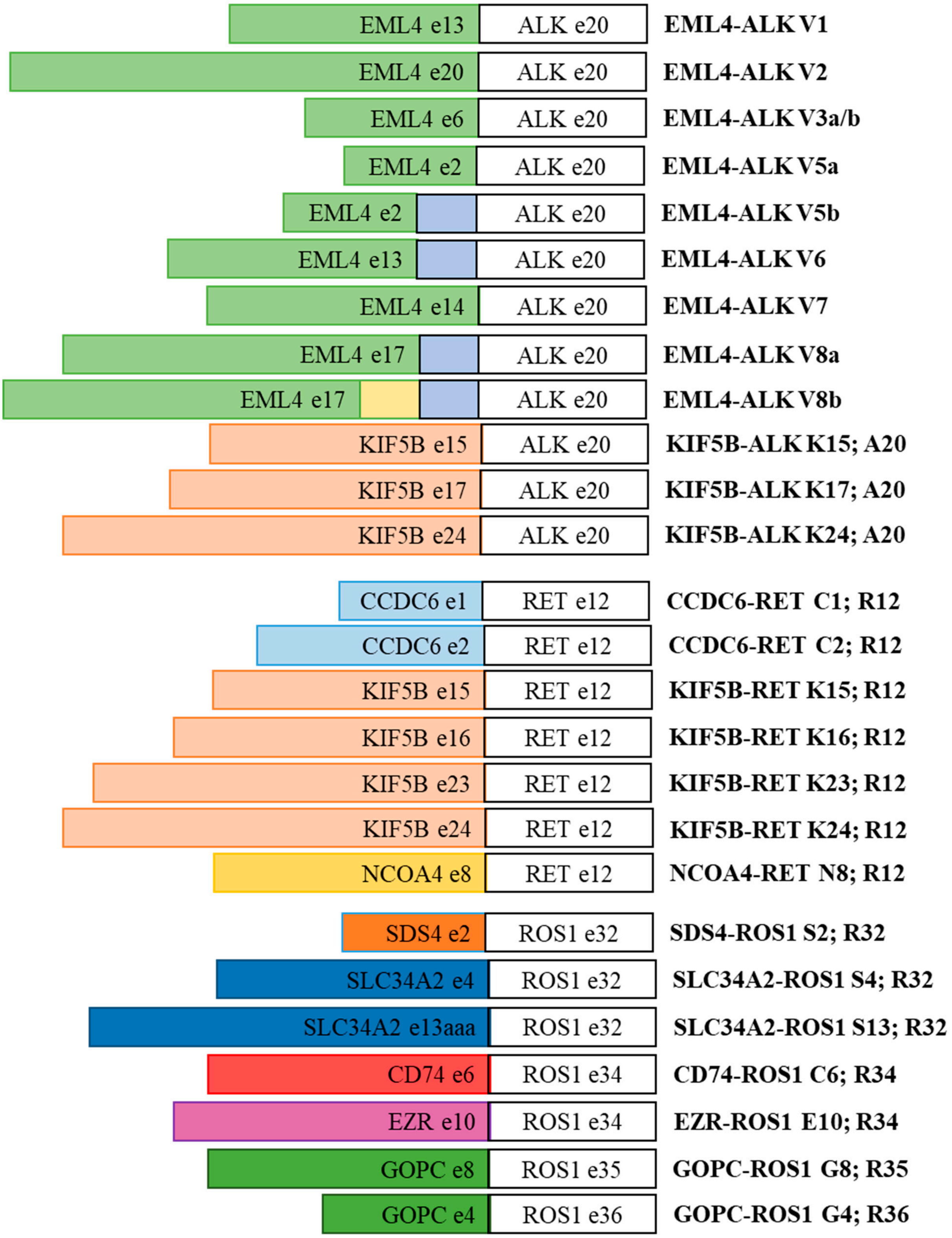

Figure S1. Schematic of Qfusion™ ALK, RET, or ROS1 fusion detection assay targets.

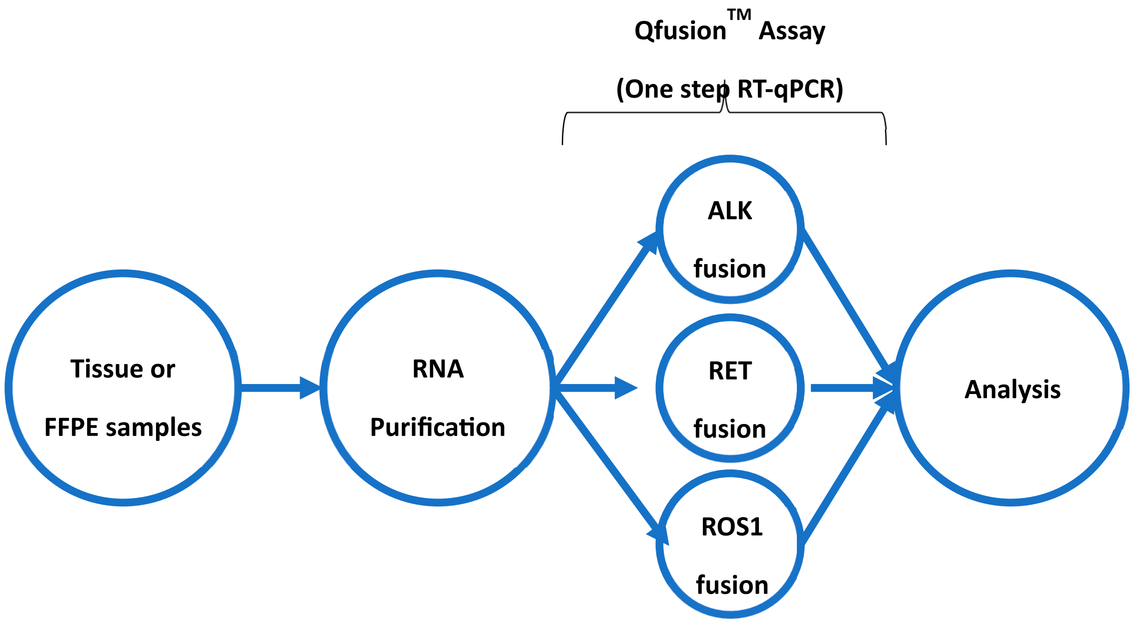

Figure S2. Schematic of RT-qPCR workflow for QfusionTM ALK, RET, or ROS1 fusion detection assay.

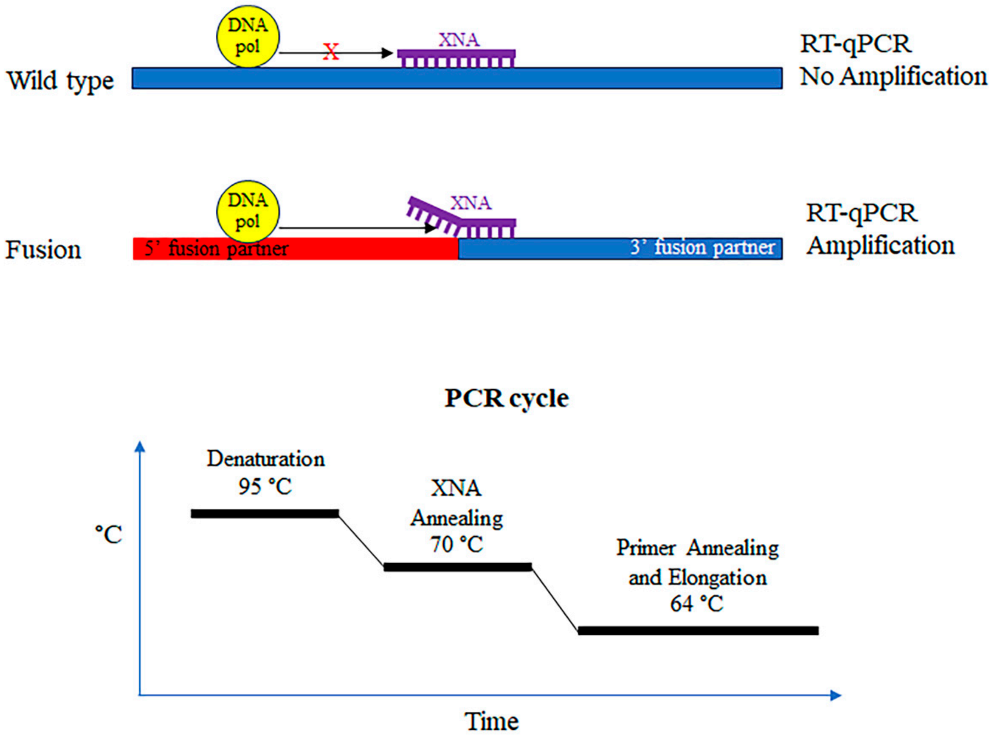

Figure S3. XNA blocker principal and PCR cycle

XNA binds tightly to wild-type sequences with a perfect match at 70°C, effectively blocking DNA polymerase during the elongation step at 64°C. Moreover, XNA can bind to fusion sequences with imperfect matches, allowing DNA polymerase to displace XNA and amplify the target sequences.

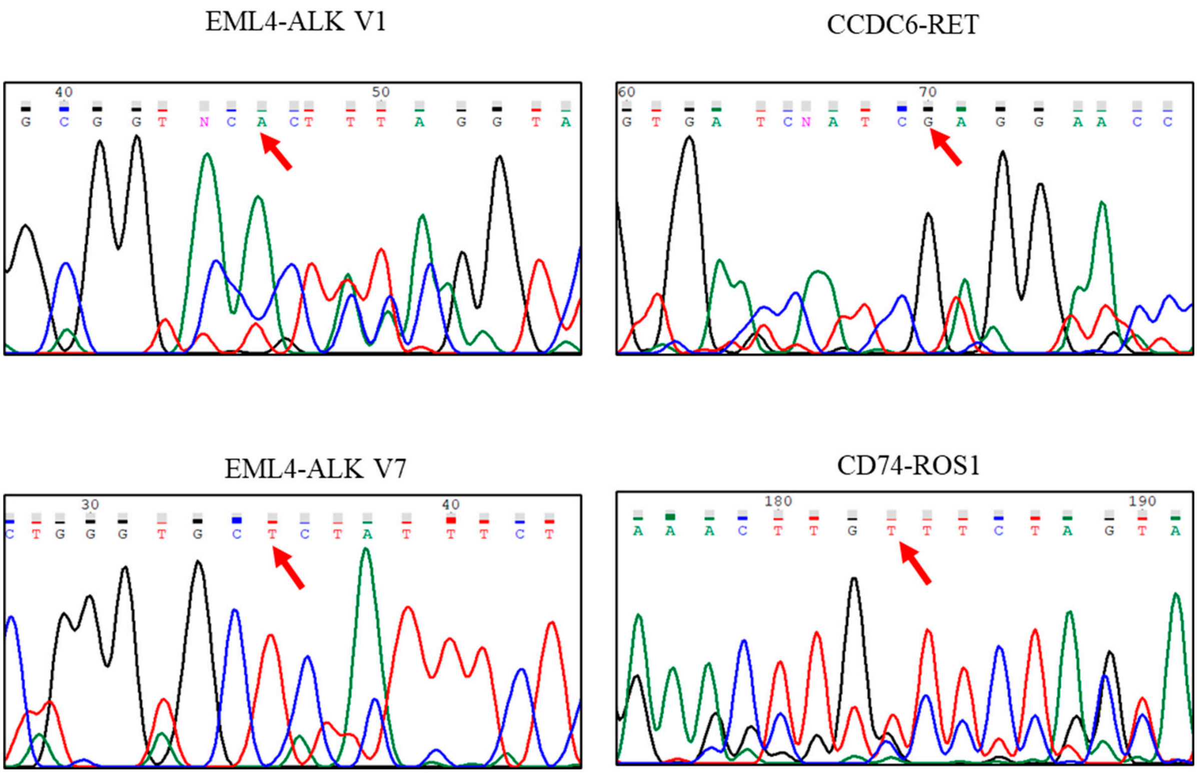

Figure S4. Confirmation of fusion genes by Sanger sequencing.
Red arrow indicates the breaking point.
